# Mutations and Copy Number Abnormalities of Hippo Pathway Components in Human Cancers

**DOI:** 10.3389/fcell.2021.661718

**Published:** 2021-06-03

**Authors:** Zhengjin He, Ruihan Li, Hai Jiang

**Affiliations:** State Key Laboratory of Cell Biology, CAS Center for Excellence in Molecular Cell Science, Shanghai Institute of Biochemistry and Cell Biology, Chinese Academy of Sciences, University of Chinese Academy of Sciences, Shanghai, China

**Keywords:** hippo deficiency, cancer formation, copy number abberation, gene mutation, cancer genome

## Abstract

The Hippo pathway is highly conserved from *Drosophila* to mammals. As a key regulator of cell proliferation, the Hippo pathway controls tissue homeostasis and has a major impact on tumorigenesis. The originally defined core components of the Hippo pathway in mammals include STK3/4, LATS1/2, YAP1/TAZ, TEAD, VGLL4, and NF2. However, for most of these genes, mutations and copy number variations are relatively uncommon in human cancer. Several other recently identified upstream and downstream regulators of Hippo signaling, including FAT1, SHANK2, Gq/11, and SWI/SNF complex, are more commonly dysregulated in human cancer at the genomic level. This review will discuss major genomic events in human cancer that enable cancer cells to escape the tumor-suppressive effects of Hippo signaling.

## Introduction

The Hippo signaling pathway is highly conserved through evolution. The core components of the pathway were originally identified in *Drosophila*. Their orthologous genes in mammals were found later (Ma et al., 2019; Snigdha et al., 2019). A large number of studies have shown that the Hippo pathway controls organ size mainly by responding to cell contact and various mechanical signals. The Hippo pathway also responds to cell polarity and G protein-coupled receptor (GPCR) signals. Given that loss of contact inhibition is one of the major hallmarks of human cancer, dysregulation of the Hippo pathway, which enables cancer cells to overcome contact inhibition, should be common in human cancers.

Gene dysregulation in human cancer can occur at various levels, including gene mutation/copy number abnormality, DNA methylation, over/under-expression, and post-translational modifications. Comparing to other types of dysregulations, mutation, and copy number abnormality data are more tractable and concrete. Therefore, this review will focus on mutation and copy number abnormality of the Hippo pathway components in human cancers.

## The Core Components of the Hippo Pathway in Cancer

The originally defined core components of the Hippo pathway include neurofibromin 2 (NF2), serine/threonine kinase 3/4 (STK3/4, originally called MST1/2), large tumor suppressor kinase 1/2 (LATS1/2), Yes1-associated transcriptional regulator (YAP1), tafazzin (TAZ), and TEA domain transcription factor (TEADS). When the Hippo pathway is activated by upstream signals, STK3/4 and Salvador Family WW domain containing protein 1 (SAV1) form a heterodimer through their C-terminal SARAH domain. Subsequently, STK3/4 phosphorylates LATS1/2, which then phosphorylates and inhibits the downstream substrate YAP1. Phosphorylated YAP1 is sequestered by 14-3-3 protein in the cytoplasm and/or degraded by the ubiquitination process.

**FIGURE 1:**
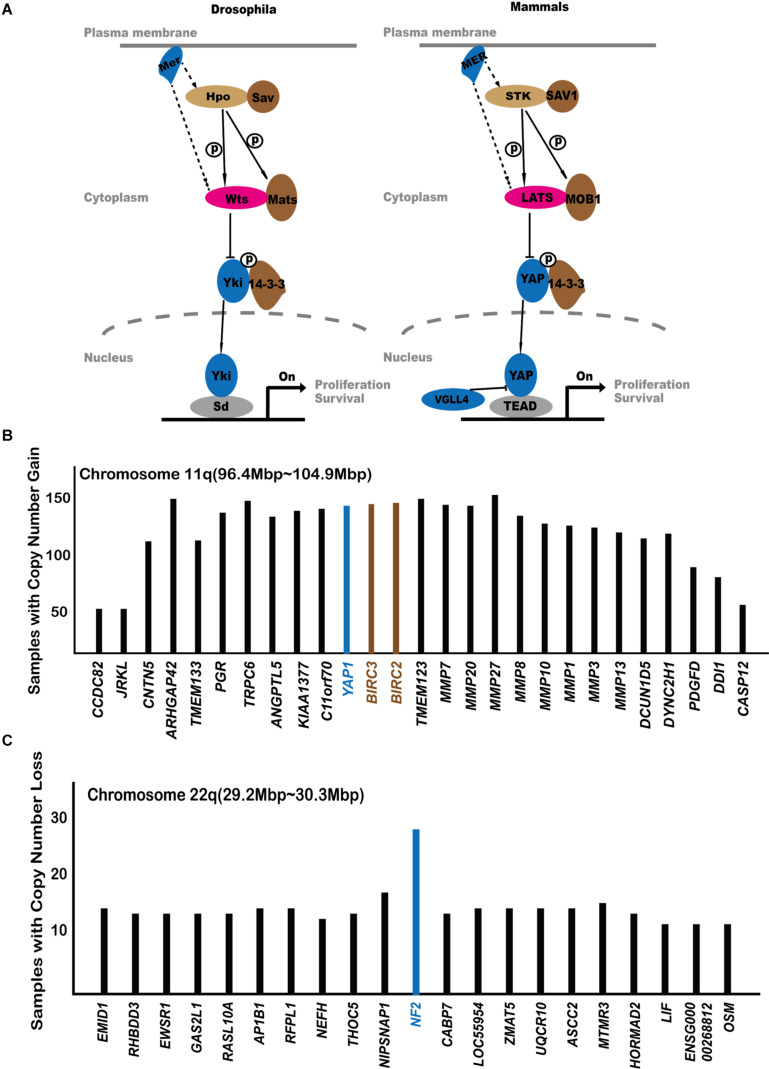
The central players of the Hippo pathway. **(A)** The originally established core components of the Hippo pathway. **(B)** Analysis of COSMIC gene amplification data with regard to the 11q22 amplicon. Genes are arranged according to their locations on chromosome 11q22. The Y axis shows how many cancer samples in COSMIC database exhibit gene amplification of each gene. If a gene is located at the amplification peak position, it is more likely to be a cancer-driving event. **(C)** Analysis of COSMIC gene amplification data with regard to the 22q deleted region.

When the Hippo pathway is inactivated, YAP1 is dephosphorylated and translocates to the nucleus, where YAP1/TAZ binds to TEAD, inducing target gene expression and promoting cell proliferation (Figure 1A). Vestigial-like family member4 (VGLL4) competitively inhibits the interaction of YAP1 and TEAD, providing another level of regulation on Hippo signaling output.

## STK3/4 and LATS1/2

In the Hippo signaling pathway, STK3/4 and LATS1/2 play antiproliferative roles and should act as tumor suppressors. However, according to the Catalogs of Somatic Mutations in Cancers (COSMIC) database^1^, genetic dysregulation of these genes is rare for most types of human cancers. This may, in part, be explained by gene redundancy (Meng et al., 2015; Plouffe et al., 2016). For example, mutation or deletion of *LATS1* alone will not enable escape from the Hippo pathway’s anti-proliferative effect since *LATS2* is still intact in cells. Therefore, single genetic events impacting *STK3/4* and *LATS1/2* will be insufficient to drive cancer formation. To fully destroy LATS1/2 activity, it will require four inactivating events to destroy both copies of *LATS1* and *LATS2*. Therefore, from a cancer development point of view, achieving YAP1 activation through inactivating Hippo kinases may not be an easy route.

In addition to gene redundancy, other possibilities may also have contributed to the low mutation rates of Hippo kinases in cancer. For example, (Moroishi et al., 2016) showed that although inactivation of *LATS1* and *LATS2* led to enhanced anchorage-independent cell growth *in vitro*, it also caused increased tumor immunogenicity and tumor regression *in vivo*. This highlights the dual functions of LATS1/2 in cancer and reflects the crucial role of Hippo kinases in regulating tissue homeostasis.

## YAP1 and TEADs

As the key component of the Hippo pathway, hyperactivation of YAP1 is widespread in cancers (Harvey et al., 2013; Mo et al., 2014) as evidenced from immunostaining of YAP1 in human cancer samples. These studies found that YAP1 is commonly enriched in the nucleus in tumors while residing in the cytoplasm in normal tissues. The percentage of cells with nuclear YAP1 staining in hepatocellular carcinomas, ovarian cancers, and non-small-cell lung cancers is 60, 15, and 65%, respectively (Harvey et al., 2013).

Yes1 Associated Transcriptional Regulator is not frequently mutated in human cancers. Although the YAP1 S127A activating mutant is commonly used in cellular studies and tumor models, there is no corresponding active point mutation of *YAP1* enriched in human cancer^2^. As discussed below, the increased activity of YAP1 in human cancers may be attributed to *YAP1* gene amplification and gene fusion as well as the dysregulation of other components of the Hippo pathway.

(Zender et al., 2006) first noticed that the chromosome 9qA1 region, containing *Yap1* gene, is amplified in mouse liver cancer cells. They also showed that overexpression of YAP1 can promote tumor formation. The corresponding amplified chromosome region in humans is 11q22 (Zender et al., 2006). This raised the possibility that the amplification of *YAP1* may contribute to cancer development. Figure 1B shows the gene amplification status in the 11q22 region, including *YAP1*. *YAP1* localized within the amplification peak region, suggesting for an oncogenic role. However, it is worth noticing that two adjacent genes, baculoviral IAP repeat containing 2/3 (*BIRC2/3*), are also amplified in human cancers. BIRC2/3 are anti-apoptotic proteins and promote cancer cell survival. It remains unclear whether the 11q22 amplicon utilizes all three genes to promote human cancer.

An interesting question is why *YAP1* amplification is not a more frequent event in human cancers. In the COSMIC database, the number of human cancer samples amplifying *YAP1* is 148. In comparison, such numbers for other major oncogenes are *MYC* = 968, *EGFR* = 514, *ERBB2* = 358, *SKP2* = 317, and *MDM2* = 324. Cellular studies demonstrated that YAP1 rapidly induces the expression of LATS2, thus forming a negative feedback loop that self-limits its activity (Moroishi et al., 2015). The YAP1 S127A mutant, which escapes negative regulation by LATS kinases, is more effective at inducing tumor and has been used in many cancer models (Zhang S. et al., 2017; Min et al., 2019; Smith et al., 2021). It is possible that, due to such a negative feedback mechanism, simple overexpression of wild-type YAP1 may not be sufficient to trigger long-lasting proliferative events, which may partially explain the relative lower frequencies of *YAP1* amplification in human cancers. On the other hand, it is possible that, similar to the case of LATS1/2 inactivation (Moroishi et al., 2016), YAP1 activity might also trigger additional events that negatively affect tumorigenesis.

Recent studies also identified *YAP1* gene fusion events in several kinds of rare cancers, such as supratentorial (ST) ependymoma (Pajtler et al., 2015, 2019), epithelioid hemangioendothelioma, cervical squamous cell carcinoma (Hu et al., 2018), endocervical adenocarcinoma (Antonescu et al., 2013), and other cancers (Picco et al., 2019; Sekine et al., 2019; Sievers et al., 2020). The fusion protein products of YAP1 in these cancers include YAP1-MAMLD1, YAP1-FAM118B, YAP1-TFE3, and YAP1-SS18. Szulzewsky et al. (2020) demonstrated that these fusion proteins are resistant to negative Hippo pathway regulation and stay in the nucleus. In addition, the fusion proteins of YAP1 are also more stable and escape from degradation.

As the final executor of Hippo signaling, the transcription factor TEAD has very low transcriptional activity without the binding of YAP1 (Ma et al., 2019). *TEAD* genes are not known to be localized in gene amplification peaks in human cancers. Mutations of *TEAD* genes are also rare in human cancer, and there are few literatures reporting the functional mutations of *TEAD* genes (Huh et al., 2019).

## NF2

Neurofibromin 2 (*NF2*) is a well-established tumor suppressor gene. It encodes Merlin, ortholog of the *Drosophila* Merlin protein, which encodes a FERM domain-containing protein. Studies of mouse and tumor patients showed that the inactivation of *NF2* is an important cause of cancer. Hamaratoglu et al. (2006) firstly found that *NF2* inhibits tumor development by regulating the Hippo pathway. Many reports demonstrated that YAP1 is dephosphorylated and activated with the loss of *NF2*, whereas the proliferation effect of *NF2* loss can be eliminated by *YAP1* knockout (Zhang et al., 2010). Hong et al. (2020) found that the lipid binding ability of Merlin is critical for its function in activating the Hippo pathway, which further clarifies the function and mechanism of *NF2* (Yin et al., 2013). Several other mouse model studies also demonstrated that deletion of *NF2* promotes tumor development (Giovannini et al., 2000; Kalamarides et al., 2002).

Hereditary loss of function mutations of *NF2* causes type 2 neurofibromatosis, a disorder characterized by neoplastic growth in the nervous system (Yin et al., 2013). Somatic loss-of-function *NF2* mutations are also found in many other kinds of cancers such as mesotheliomas and bladder, thyroid, and skin cancer. The COSMIC database indicates that *NF2* mutations are highly enriched for nonsense mutations^3^. *NF2* is also located at a deletion peak in cancer samples (Figure 1C). This indicates that gene mutation and gene deletion are both common means of *NF2* loss of function in human cancers.

## VGLL4

Vestigial Like Family Member 4 can inhibit organ overgrowth and cancer formation caused by YAP1 dysregulation in both human and *Drosophila* (Guo et al., 2013; Zhang et al., 2014). Jiao et al. (2014) found that VGLL4 competes with YAP1 to bind TEADs. Such an event will inhibit gene transcription by YAP1–TEADs and suppress cell proliferation (Zhang Y. et al., 2017).

Vestigial Like Family Member 4 has been described as a tumor suppressor in many cancers (Jiao et al., 2014; Zhang et al., 2014; Zhang Y. et al., 2017; Gallagher et al., 2020). For example, (Zhang et al., 2014) found that the VGLL4 expression level in mouse and human lung tumor specimen is significantly lower than in normal tissue. Overexpression of VGLL4 inhibits the progression of lung cancer in mice (Zhang et al., 2014). The findings of Jiao et al. (2014); Zhang Y. et al. (2017) in gastric cancer and breast cancer also support the idea that *VGLL4* inhibits tumor progression.

Although *VGLL4* is rarely mutated in human cancer, it is located at the short arm (3p) of chromosome 3, which is lost in many types of cancer (Williamson, 2002; Cancer Genome Atlas Research Network, 2013; Jonasch et al., 2020; Nidorf et al., 2020; Shaikh et al., 2020). The Cancer Genome Atlas (TCGA) Research Network found that around 90% of clear cell renal carcinoma patients exhibit loss of one or both copies of chromosome 3p. Chromosome 3p loss is also commonly observed in lung and stomach cancers. The rate of both 3p arm loss is lower in human cancer, typically around 10–20% in renal clear cell carcinoma. It is worth noticing that, through 3p loss, cancer cells also delete other important tumor suppressors such as *VHL*, *SETD2*,*BAP1*, and *PBRM1*. These events may also promote cancer development independent of *VGLL4*.

## Other Components of the Hippo Pathway in Cancer

In addition to the afore-mentioned core components of the Hippo pathway, several recent studies identified new regulators of Hippo signaling. Some of these new regulators are also prominently dysregulated in human cancers, providing additional routes for cancer cells to escape from Hippo signaling.

## FAT1

FAT atypical cadherin 1 (FAT1) is a transmembrane protein, homologous to the tumor-suppressor genes *fat* and *kujelei* (also known as *fat2*) in *Drosophila*. By analyzing the TCGA database, (Martin et al., 2018) found that *FAT1* shows a high-frequency mutation in many types of cancer. The study revealed that the cytoplasmic domain of FAT1 can activate the Hippo pathway by recruiting Hippo components such as NF2, STK3/4, and LATS1/2 to the cell membrane, forming a “kinase signalome” (Martin et al., 2018). In addition, other researchers also found that FAT1 regulates the Hippo pathway by YAP1 and TAZ (Ahmed et al., 2015; Li et al., 2018). One recent study showed that deletion of *FAT1* in mouse epithelial cells induces epithelial–mesenchymal transition of epithelial cells and promote tumorigenesis in mice (Pastushenko et al., 2021).

**FIGURE 2:**
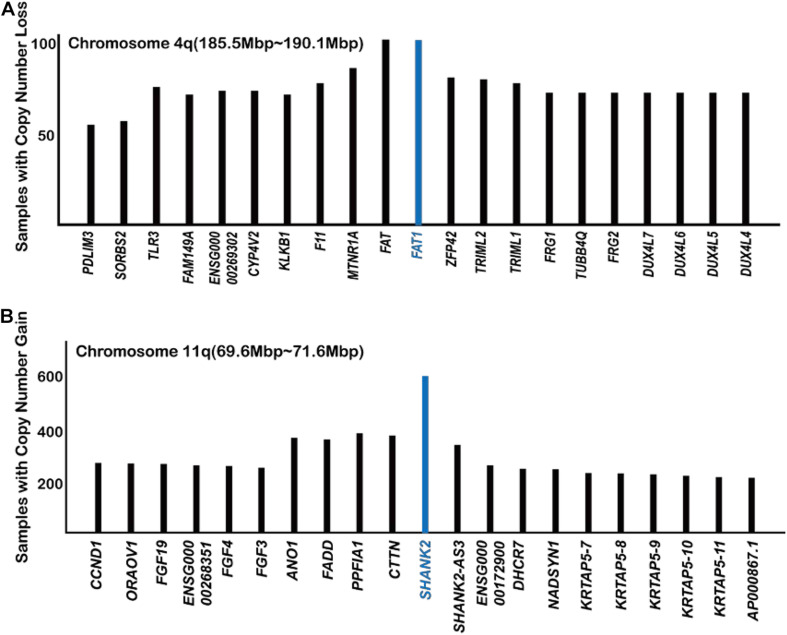
Analysis of COSMIC gene copy number data with regard to FAT1 **(A)** and SHANK2 **(B)** (https://cancer.sanger.ac.uk/cosmic/).

According to the TCGA database, *FAT1* is frequently mutated in many types of cancer (Katoh, 2012; Ahmed et al., 2015; Zhang et al., 2016). *FAT1* exhibits 25.2 and 25.3% mutation rates in head and neck cancer and uterine corpus endometrial carcinoma, respectively, according to the TCGA database. Analysis of the COSMIC database indicated that *FAT1* is located at a gene deletion peak in tumor samples, further suggesting a tumor suppressor role for FAT1 (Figure 2A).

## SHANK2

The SH3 and multiple ankyrin repeat domains (SHANK) protein family has been studied in the field of neuroscience mostly. As a scaffold protein, SHANK promotes synapse formation and balances synaptic transmission. In a recent study (Xu et al., 2020), found that the overexpression of SHANK2 ortholog in *Drosophila* suppresses Hippo signaling, causing an overgrowth of wings and eyes. In human cells, overexpression of SHANK2 also caused sustained YAP1 activity even at high cell density and promoted tumor formation in mice. SHANK2 inhibits the LATS1/2-mediated phosphorylation of YAP1 by competing for binding to rho guanine nucleotide exchange factor 7 (ARHGEF7), which is an activator protein of LATS1/2 (Heidary Arash et al., 2014). Analysis of the COSMIC database also showed that *SHANK2* is the most frequently amplified gene in the 11q13 amplicon (Figure 2B; Xu et al., 2020). It is worth noticing that the number of cancer samples with *SHANK2* amplifications way exceeds those with *YAP1* amplification, *FAT1* deletion, and *NF2* deletion (Figures 1B, 2), indicating a previously unnoticed role of *SHANK2* as a major oncogene.

## Gq/G11

The G protein subunit alpha Q (GNAQ) and G protein subunit alpha 11 (GNA11) genes encode Gq and G11 proteins, respectively, which play an essential role in GPCR signaling pathway. Yu et al. (2014) found that the mutant Gq/G11 can activate the YAP1 protein. In addition, inhibition of YAP1 can block the proliferation of Gq/G11 mutant cells These results imply that the pro-proliferation effect of mutant Gq/G11 depends on the function of YAP1.

Previous studies (Van Raamsdonk et al., 2009, 2010) showed that overexpression of mutant Gq/G11 causes normal melanocyte transformation, whereas knockdown of *Gq/G11* blocks tumor formation in xenograft experiments. Data from the TCGA database indicated that the percentage of *GNAQ* and *GNA11* gene mutation is 50 and 43.8%, respectively, in uveal melanoma patients, suggesting a major involvement of the Hippo pathway for this type of cancer.

## SWI/SNF

SWI/SNF is a multi-subunit ATP-dependent chromosome remodeling complex. The SWI/SNF complex plays key roles in regulating gene expression and tissue homeostasis. Mutations of the subunits of this complex are detected in a variety of human malignancies (Kadoch et al., 2013; Shain and Pollack, 2013; Lou et al., 2020).

Increasing evidence indicated that the SWI/SNF complex inhibits tumor development (Weissman and Knudsen, 2009; Wilson and Roberts, 2011; Shain and Pollack, 2013; Ribeiro-Silva et al., 2019). A large number of studies showed that the deletion of components of SWI/SNF promotes tumor development in mouse models (Klochendler-Yervin et al., 2000; Roberts et al., 2000; Glaros et al., 2007, 2008). In a recent study, (Chang et al., 2018) found that the ARID1A subunit of the SWI/SNF complex binds to and inactivates YAP1. Therefore, once the SWI/SNF complex is inactivated by various mutations in cancer cells, YAP1 will be released and promote carcinogenesis. In addition, the SWI/SNF complex may also inhibit cancer development by maintaining genome stability (Ribeiro-Silva et al., 2019). The statistical results of Ribeiro-Silva et al. (2019) indicate that mutations in genes encoding for SWI/SNF subunits are found in approximately 20% of all human cancers of various types. This may constitute one of the most frequent routes of Hippo dysregulation in human cancer.

## Conclusion

The Hippo pathway, by responding to cell density and maintaining cell–cell contact, is a crucial barrier for tumor development. When the Hippo pathway is dysregulated, cells will acquire the potential for uncontrolled proliferation, promoting cancer formation. Although mutations and/or copy number abnormalities directly impacting the core Hippo kinases are relatively rare in human cancers, cancer cells manage to escape from Hippo regulation by means of other upstream and downstream Hippo regulators, including FAT1, SHANK2, SWI/SNF, Gq/11, VGLL4, *etc*. In this review, we focused on Hippo dysregulation in human cancers at the genomic level. It is also worth noticing that other oncogenic events, for example, IDH1 mutation, can also affect the Hippo pathway components through gene hypermethylation (Gu et al., 2020).

**FIGURE 3:**
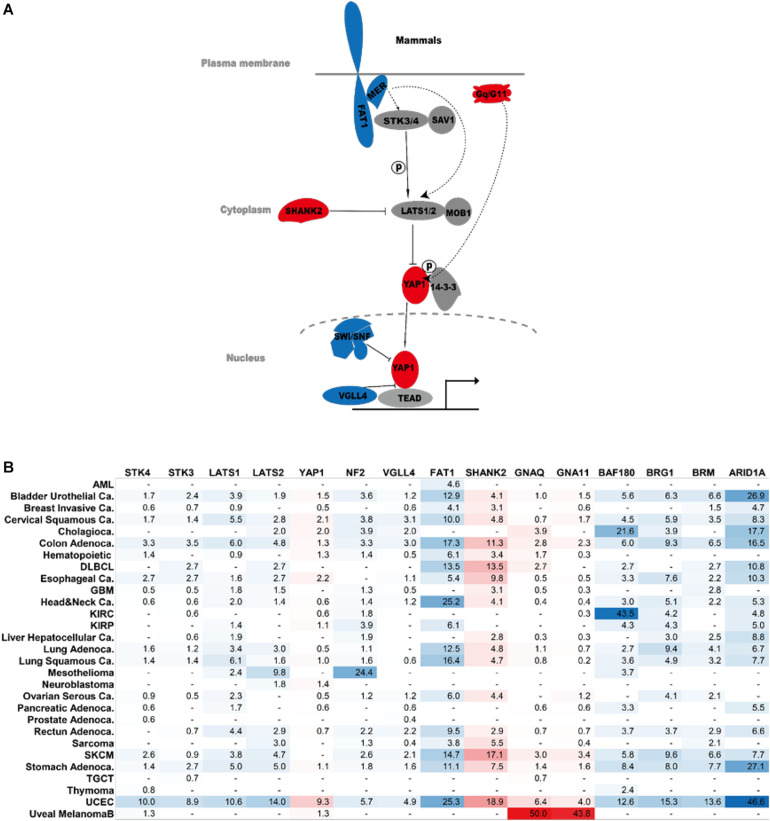
Hippo components in human cancer. **(A)** Major cancer players of the Hippo signaling pathway. Genes in red are oncogenes. Genes in blue are tumor suppressors. **(B)** Percentage of genetic abnormalities of the indicated Hippo pathway components in human cancer. The analysis was performed on The Cancer Genome Atlas dataset. AML, acute myeloid leukemia; DLBCL, diffuse large B cell lymphoma; GBM, glioblastoma multiforme; KIRC, kidney renal clear cell carcinoma; KIRP, kidney renal papillary cell carcinoma; SKCM, skin cutaneous melanoma; TGCT, testicular germ cell tumors; UCEC, uterine corpus endometrial carcinoma.

Several recent studies also identified other important regulators of the Hippo pathway, including the RAP family of small GTPase, MST4, and others (Meng et al., 2018; An et al., 2020). These genes do not appear to be frequently dysregulated at the genomic level in human cancers, possibly due to gene redundancy or other reasons. For example, all three *RAP2* genes (*Rap2A/B/C*) need to be simultaneously knocked out to cause YAP1 nuclear localization (Meng et al., 2018). Therefore, these genes are not included in this review. Figure 3 summarizes the major cancer players of the Hippo pathway and their frequency of genetic dysregulation in various forms of human cancers. Such findings will help bring a clearer view of Hippo pathway dysregulation in cancers as well as point to potential precision medicine approaches targeting YAP1 activity for cancer therapy.

For example, cancers that dysregulate the Hippo pathway may be more sensitive to suppression of YAP1 activity. On the one hand, the degradation of YAP1 may be a choice for cancer treatment. Proteolysis-targeting chimera (PROTAC) technology could provide us with a useful method for YAP1 degradation (Li and Song, 2020). On the other hand, VGLL4’s ability to disrupt YAP1–TEADs interaction indicates that peptide mimics of VGLL4 could potentially be useful for suppressing cancers driven by Hippo pathway dysregulation. Newly identified oncogenes in the Hippo pathway such as Gq/G11 and SHANK2 may also represent potential PROTAC targets for cancer treatment.

## Author Contributions

All authors wrote the manuscript. ZH prepared Figures.

## Conflict of Interest

The authors declare that the research was conducted in the absence of any commercial or financial relationships that could be construed as a potential conflict of interest.

## Footnotes

1 https://cancer.sanger.ac.uk/cosmic

2 https://cancer.sanger.ac.uk/cosmic/gene/analysis?ln=YAP1

3 https://cancer.sanger.ac.uk/cosmic/gene/analysis?ln=NF2
